# LUC7L3/CROP inhibits replication of hepatitis B virus via suppressing enhancer II/basal core promoter activity

**DOI:** 10.1038/srep36741

**Published:** 2016-11-18

**Authors:** Yuan Li, Masahiko Ito, Suofeng Sun, Takeshi Chida, Kenji Nakashima, Tetsuro Suzuki

**Affiliations:** 1 Department of Virology and Parasitology, Hamamatsu University School of Medicine, Shizuoka 431-3192, Japan

## Abstract

The core promoter of hepatitis B virus (HBV) genome is a critical region for transcriptional initiation of 3.5 kb, pregenome and precore RNAs and for the viral replication. Although a number of host-cell factors that potentially regulate the viral promoter activities have been identified, the molecular mechanisms of the viral gene expression, in particular, regulatory mechanisms of the transcriptional repression remain elusive. In this study, we identified LUC7 like 3 pre-mRNA splicing factor (LUC7L3, also known as hLuc7A or CROP) as a novel interacting partner of HBV enhancer II and basal core promoter (ENII/BCP), key elements within the core promoter, through the proteomic screening and found that LUC7L3 functions as a negative regulator of ENII/BCP. Gene silencing of LUC7L3 significantly increased expression of the viral genes and antigens as well as the activities of ENII/BCP and core promoter. In contrast, overexpression of LUC7L3 inhibited their activities and HBV replication. In addition, LUC7L3 possibly contributes to promotion of the splicing of 3.5 kb RNA, which may also be involved in negative regulation of the pregenome RNA level. This is the first to demonstrate the involvement of LUC7L3 in regulation of gene transcription and in viral replication.

Hepatitis B virus (HBV) infection is a major cause of acute and chronic hepatitis and is closely associated with development of cirrhosis and hepatocellular carcinoma (HCC) worldwide. HBV is a small enveloped virus with a partially double-stranded DNA genome. The 3.2 kb HBV genome transcribes four major species of unspliced RNA transcripts; 3.5, 2.4, 2.1 and 0.7 kb from the core, preS1, preS2/S and X promoters, respectively, as well as a variety of spliced viral RNAs. Core promoter produces 3.5-kb transcripts; the pregenome and precore RNAs (termed as pgRNA in this study). HBV pgRNA not only serves as the mRNA that codes for core and polymerase proteins but can be packaged into nucleocapsid as a pregenomic RNA intermediate. Two enhancers; ENI and ENII are also involved in modulation of the viral transcription basically via up-regulating the activities of the HBV promoters. The transcriptional regulation of HBV genes is prerequisite for the control of the viral replication^1^,^2^,^3^. In contrast, functional roles and significance of spliced RNAs in HBV lifecycle remain largely unclear.

The core promoter and ENII are located in the region that overlaps with the X gene. The core promoter can be divided into two elements; basal core promoter (BCP)(nucleotide [nt] 1743–1849) sufficient to initiate transcription and an upper regulatory region (nt 1643–1742). The latter region is composed of the core upstream regulatory sequence (CURS) that stimulates the BCP and of negative regulatory element (NRE). ENII is located at nt 1685–1773, immediately upstream of the BCP and overlaps with the CURS of the core promoter. ENII stimulates the viral promoter activities in the manner independent (preS1, preS2/S and X) or dependent (BCP) on position/orientation. ENII function is the results of various interactions with liver-enriched or ubiquitous transcription factors including the nuclear receptor superfamily. Nevertheless, further studies to elucidate regulatory mechanisms for accurate HBV gene modulation on the complex interplay between cellular factors and their corresponding sites within these critical elements are required^2^,^3^,^4^.

The course of chronic HBV infection frequently leads to alteration of the viral genome. Several nucleotide mutations that naturally occurred in the ENII/BCP region have been shown affecting viral function in cultured cells^5^,^6^,^7^,^8^,^9^. It is assumed that ENII/BCP mutations potentially affect binding between the region and cellular factors involved in the transcriptional regulation, leading to less production of the 3.5-kb mRNA and HBe antigen. Although effects of ENII/BCP mutations on the development of HCC has been poorly investigated, it has been reported that mutations within the region, for instance, T1762/A1764 in the BCP are often found in patients with advanced liver diseases such as HCC^10^,^11^,^12^,^13^.

In this study, to further understand the molecular mechanisms underlying pivotal roles of ENII/BCP in HBV replication and possibly in the viral pathogenesis, we employed a proteome-based screening to identify candidate proteins interacting with ENII/BCP of the HBV genome. One of the identified proteins, LUC7 like 3 pre-mRNA splicing factor (LUC7L3, also known as hLuc7A or CROP) was found to be a negative regulator of ENII/BCP activity. The silencing or overexpression of LUC7L3 regulated HBV replication. Ectopic expression of the siRNA-resistant LUC7L3 apparently restored down-regulation of the core promoter activity mediated by the gene silencing. Mutational analyses demonstrated that the N-terminal zinc finger motif within LUC7L3 is involved in the negative regulation of pgRNA expression and that the nt 1666–1700 region in ENII is important for LUC7L3-mediated negative regulation of the ENII/BCP activity. In addition, it is likely that LUC7L3 plays a role in promotion of the pgRNA splicing. Our data suggested a role of LUC7L3 as a novel negative regulator in HBV replication process.

## Results

### Identification of nuclear proteins that interact with the enhancer II/basal core promoter region and control the HBV core promoter activity

**Figure 1:**
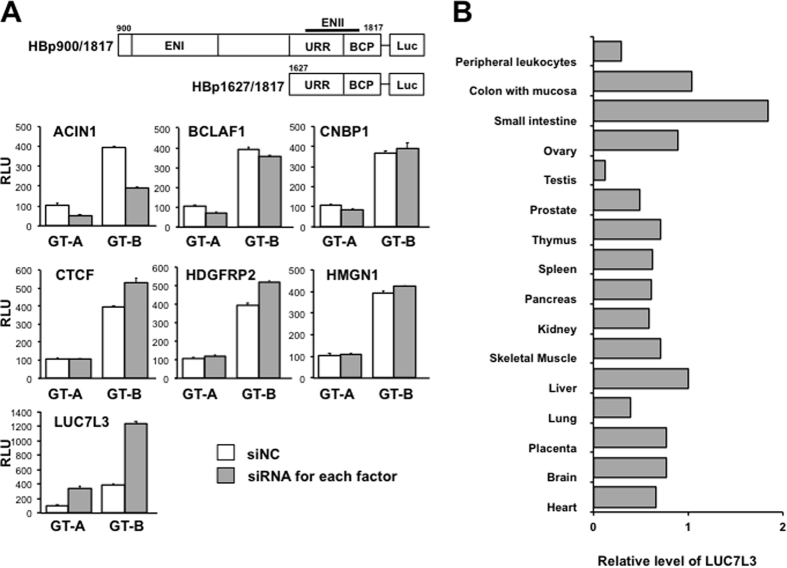
Effect of knockdown of ENII/BCP-interacting factors on the viral core promoter and distribution of LUC7L3 mRNA expression in human tissues. (**A**) The luciferase reporter constructs harboring HBV cis-acting elements for the entire core promoter (pGLHBp900/1817) and for the ENII/BCP region (pGLHBp1627/1817) with numbering nucleotide positions used are depicted (upper). HuH7 cells introducing siRNA for each ENII/BCP-interacting factor identified by the proteomics screening (siRNA for each factor) or its negative control (siNC) were transfected with pGLHBp900/1817, in which the viral sequence is derived from genotype A (GT-A) or B (GT-B) of HBV. Two days later, cells were harvested and used for the reporter luciferase assays. Values (RLU) are normalized with the values of transfection with control siRNAs and GT-A reporter, set at 100%. Values shown represent the means with standard errors of the means (SEM) from three independent transfection of siRNA. (**B**) Expression of LUC7L3 gene in various human tissues was assessed by quantitative PCR using the tissue-specific human cDNA panel. Values shown were normalized by those of RNA polymerase II gene expression. The relative value for liver was set at 1.

To identify novel host factors which are involved in regulating the transcription of 3.5-kb pregenome/precore RNA (pgRNA) of HBV, we first carried out proteomics screening in which nuclear proteins binding to biotinylated capture DNA of HBV sequence covering enhancer II and basal core promoter (ENII/BCP)(nt 1627–1817) were isolated using the magnetic microbeads separation system (Supplementary Table S1). Among 89 proteins identified by LC-MS/MS analysis, seven proteins; ACIN1, CNBP1, LUC7L3, BCLAF1, CTCF, HDGFRP2, HMGN1, were selected because of their involvement in transcription and RNA metabolism, followed by testing as to whether they play roles in the HBV gene expression. The effect of siRNA-based gene silencing of each candidate on the activity of HBV core promoter was assessed. After 24 h of siRNA transfection, cells were transfected with the reporter luciferase plasmids, followed by measuring luciferase activities after further 48-h culture. We found that LUC7L3 knock-down led to marked increase in the promoter activities derived from both genotypes A and B (Fig. 1A).

LUC7L3, a human homolog of yeast U1 snRNP-associated factor, is known as a nuclear protein that is possibly involved in pre-mRNA splicing^14^,^15^. Expression of LUC7L3 gene in human organs was determined by RT-quantitative PCR (RT-qPCR). Although LUC7L3 expression was detected in most of organs tested, the highest expression was observed in the small intestine and the relatively high levels were found in the liver, ovary and colon (Fig. 1B).

### Negative regulation of enhancer II/basal core promoter activity and HBV replication by LUC7L3

**Figure 2:**
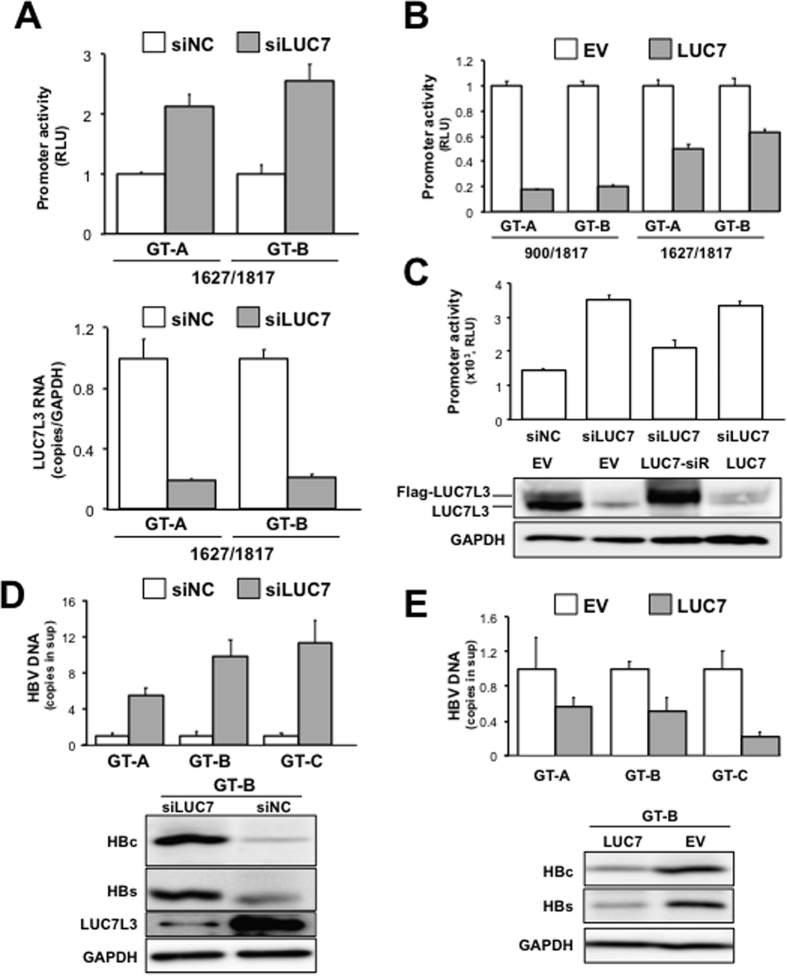
Negative regulation of ENII/BCP activity and HBV replication by LUC7L3. (**A**) Effect of LUC7L3 knockdown on HBV ENII/BCP activity was determined. At 48 h after introducing LUC7L3 siRNAs (siLUC7) or its negative control (siNC), HuH7 cells were transfected with pGLHBp1627/1817 whose HBV sequence is derived from genotype A (GT-A) or B (GT-B), followed by further 48-h culture. Cells were used for the luciferase assay as indicated in Fig. 1A (upper) and for determination of LUC7L3 mRNA expression by RT-qPCR (lower). (**B**) Effect of over-expression of LUC7L3 on HBV promoter activities was tested. HuH7 cells were transfected with the LUC7L3-expression plasmid (LUC7) or an empty vector (EV) together with pGLHBp900/1817 (900/1817) or pGLHBp1627/1817 (1627/1817). Cells were harvested two days later to determine the luciferase activities. (**C**) Restoration of the promoter activity by expression of siRNA-resistant LUC7L3 was shown. HuH7 cells were transfected with LUC7L3 siRNAs (siLUC7) or its negative control (siNC) together with LUC7L3-expression plasmids (LUC7) or siRNA-resistant LUC7L3 (LUC7-siR). After 24 h, cells were further transfected with pGLHBp900/1817 for evaluating the luciferase activities. Expression of LUC7L3 proteins and GAPDH was assessed by immunoblotting (lower). (**D**) Effect of LUC7L3 knockdown on HBV replication and production of the viral proteins were determined. At 48 h after introducing LUC7L3 siRNAs (siLUC7) or its negative control (siNC), cells were transfected with pUC-HB-Ae, -Bj or -CAT, which carries a 1.24-fold HBV genome derived from genotype (**A**–**C**), respectively, followed by 48-h culture. The supernatants and cells were subjected to qPCR of the particle-associated HBV DNA (upper) and immunoblotting of HBs and HBc antigens, LUC7L3 and GAPDH (lower), respectively. (**E**) Effect of over-expression of LUC7L3 on HBV replication and production of the viral proteins were determined. HuH-7 cells were transfected with the LUC7L3-expression plasmid (LUC7) or an empty vector (EV) together with pUC-HB-Ae, -Bj or -CAT. At 72 h post-transfection, the supernatants and cells were harvested to determine the particle-associated HBV DNA (upper) and the viral antigens (lower) as indicated in (**D**). Values shown represent the means with SEM obtained from three independent transfection of siRNAs or plasmids.

The functional relevance of LUC7L3 on transcriptional regulation of pgRNA was further assessed. Knockdown of LUC7L3 resulted in significant increase in the ENII/BCP activity, likewise the activity of entire core/pregenome promoter (Fig. 2A; upper panel). Knockdown efficiencies were confirmed by RT-qPCR (Fig. 2A; lower panel). Effect of over-expression of LUC7L3 on the promoter activities was also determined. After 48 h of co-transfection with the LUC7L3-expression plasmid and the reporter plasmid for entire core promoter or ENII/BCP, luciferase activities in cell lysates were measured. As expected, both activities for the entire core promoter and ENII/BCP were significantly reduced in cells transfected with the LUC7L3-expression plasmid (Fig. 2B). Effect of the gene silencing of LUC7L3 by siRNA was apparently restored by ectopic expression of the siRNA-resistant LUC7L3 but not by that of wild-type LUC7L3 (Fig. 2C), confirming that the knockdown-mediated increase in the core promoter activity observed was not due to the off-target effect by siRNA used.

Next we investigated loss- and gain-of-functions of LUC7L3 with respect to the replication and production of HBV. After 48 h of siRNA transfection, cells were transfected with a plasmid carrying a 1.24-fold HBV genome derived from genotype A, B or C (pUC-HB-Ae, -HB-Bj or –HB-CAT). Further 48-h culture later, the supernatants and cells were harvested and used to determine the particle-associated HBV DNA by qPCR and HBs and HBc antigens by immunoblotting, respectively. The viral DNA levels in the supernatants were 5- to 9-fold higher in the cultures with knockdown of LUC7L3 compared with those with introducing negative control siRNA (Fig. 2D, upper panel). Steady-state levels of HBs and HBc antigens in cells with LUC7L3 knockdown were obviously higher compared to the control cells (Fig. 2D, lower panel). In contrast, over-expression of LUC7L3 in cells replicating the HBV genome resulted in reduced expression of the viral RNA and antigens (Fig. 2E), indicating that increased expression of LUC7L3 potentially leads to the opposite effect on the HBV replication as observed in case of the gene silencing. It is noted that neither over-expression nor knockdown of LUC7L3 exhibited an influence on the cell viability under the experimental conditions used (Supplementary Fig. S1).

### A key role of the N-terminal zinc finger motif within LUC7L3 in the negative regulation of expression of HBV pgRNA

**Figure 3:**
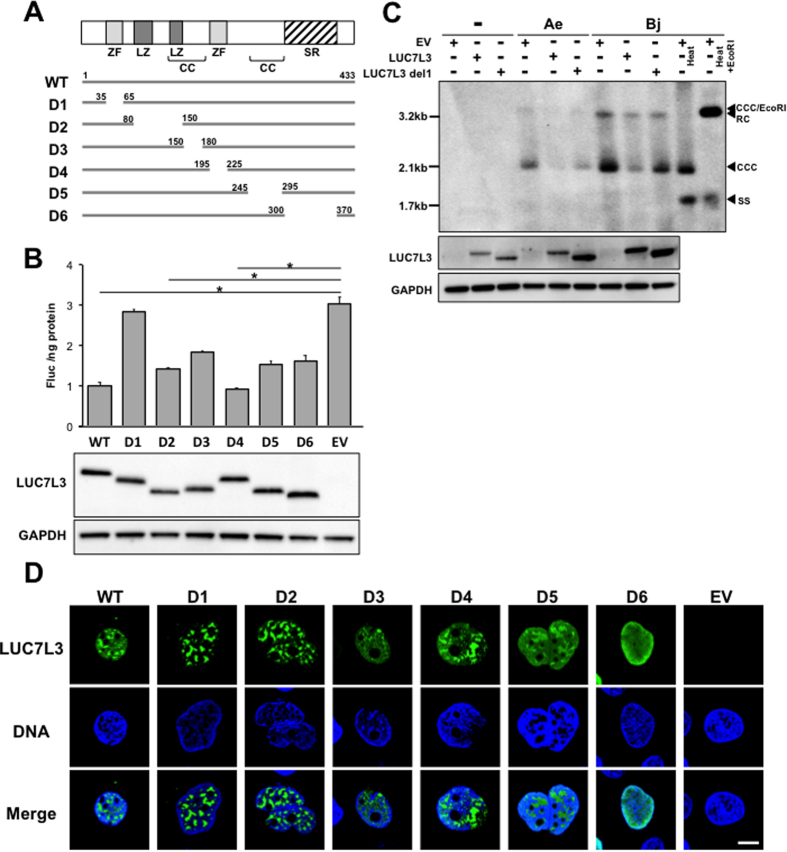
Effects of deletions within LUC7L3 on its subcellular distribution and on expression of the viral pgRNA. (**A**) A series of deletion mutants of LUC7L3 used in this study were indicated. A schematic diagram of predicted domain structures of LUC7L3 is indicated at the top. ZF, zinc-finger domain; LZ, leucine zipper domain; SR, SR-rich domain; CC, coiled-coil domain. (**B**) HuH7 cells were transfected with FLAG-tagged LUC7L3 (LUC7L3) or its partial deletions (D1–D6) or empty vector (EV) together with pGLHBp1627/1817 (1627/1817). Cells were harvested three days later to determine the luciferase activities (upper) and expression of LUC7L3 proteins as well as of GAPDH was assessed by immunoblotting (lower). (**C**) HuH7 cells were transfected with FLAG-tagged LUC7L3 (LUC7L3) or its partial deletion (LUC7L3 D1) or empty vector (EV) together with pUC-HBAe, -Bj or pUC19 (−). At 5 days post-transfection, Hirt DNA was prepared from the transfected cells and subjected to Southern blot analysis. RC, rcDNA; CCC, cccDNA; SS, single-strand DNA. cccDNA was resistant to denaturation at 85 °C for 5 min (Heat), but sensitive to EcoRI digestion (Heat + EcoRI). (**D**) Subcellular localization of LUC7L3 and its deletion mutants were determined by confocal microscopy images of cells transfected with expression plasmids for FLAG-tagged LUC7L3 (LUC7L3) or its partial deletions (D1–D6) or empty vector (EV). Cells were immunostained with anti-FLAG antibody as a first antibody (LUC7L3), and counterstained with Hoechst 33342 to label nuclei (DNA). Scale bar, 10 μm. Statistical significances compared with EV were shown. *p < 0.05, Student’s t test.

As represented in Fig. 3A (upper), LUC7L3 contains two cysteine/histidine zinc finger motifs in its N terminus and central region (ZF1, ZF2) and two leucine zipper-like repeats in the N-terminal half (LZ1, LZ2). Its C-terminal half is extremely hydrophilic and contains the arginine/serine-rich domain (RS) that has been observed in RNA splicing factors. To define the region in LUC7L3 responsible for its effect on the pgRNA expression, we constructed a series of LUC7L3 deletion mutant plasmids; pCAG-LUC7L3-D1~-D6, each expressing LUC7L3 that lacks one of the domains/motifs mentioned above or coiled-coil domains (CC1, CC2), with a FLAG tag (Fig. 3A). Effects of over-expression of each LUC7L3 deletion mutant on the viral RNA level were assessed in cells co-transfected with pUC-HB-Bj (Fig. 3B). Among mutants tested, expression of LUC7L3-D1 exhibited no or only a limited influence on the ENII/BCP activity and the pgRNA level, demonstrating that the N-terminal zinc finger motif (ZF1), which is the C3H-type (three cysteines and one histidine), is important for the negative regulation of the ENII/BCP activity mediated by LUC7L3. To determine a role of LUC7L3 in HBV replication, HuH7 cells were transfected with pUC-HB-Bj together with either expression construct for wild-type LUC7L3 or LUC7L3-D1, subjecting to Southern blotting at 5 days post-transfection (Fig. 3C). As expectedly, the levels of intracellular HBV DNA, in particular, a possible form of covalently closed circular DNA (cccDNA) was markedly lower in cells expressing wild-type LUC7L3. A lower level of the cccDNA was also seen in cells expressing LUC7L3-D1 but its difference compared to the level in control cells was limited.

The subcellular distribution of LUC7L3 and its mutants was also determined (Fig. 3D). As previously reported, LUC7L3 was detected throughout the nucleus with a speckled staining pattern. In contrast to LUC7L3-D1 and -D2 being more exclusively granular nuclear patterns, LUC7L3-D6 showed a homogeneous nuclear distribution, found largely in the nucleoplasm. It is likely that several motifs or domains, including ZF1, known to be involved in DNA/RNA binding potentially contribute to subcellular localization of LUC7L3.

### The nt 1666–1700 region within enhancer II/basal core promoter is important for its negative regulation mediated by LUC7L3

**Figure 4:**
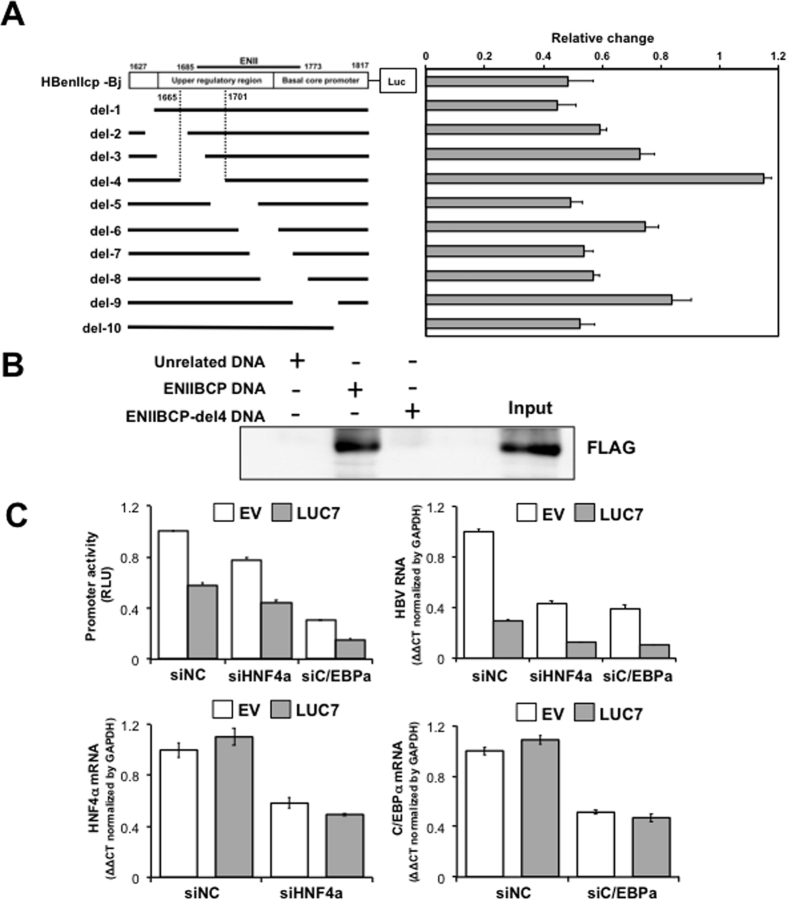
Effects of deletions within the ENII/BCP sequence on down-regulation of the ENII/BCP activity mediated by LUC7L3. (**A**) Deletion constructs of the ENII/BCP region of the HBV genome linked to the firefly luciferase gene are shown. Cells were co-transfected with one of these reporter constructs and the LUC7L3-expressing plasmid or an empty vector. At 48 h post-transfection, cells were harvested and subjected to the luciferase assay. Values obtained without LUC7L3 expression were set as 1 for expression of each reporter. Results represent the means with SEM from three independent transfectants. (**B**) Interaction of LUC7L3 with the ENII/BCP sequence but not with its deletion, del4 as indicated in (**A**), was shown by DNA pull-down assay. *In vitro* synthesized biotinylated DNA derived from entire ENII/BCP or its deletion of nt 1666–1700 region was mixed with the nuclear extract prepared from cells transfected with LUC7L3 construct. Biotinylated DNA-protein complexes were captured with streptavidin-conjugated beads, followed by immunoblotting with anti-FLAG antibody. (**C**) Effects of knockdown of HNF4α or C/EBPα on down-regulation of the ENII/BCP activity and the pgRNA expression were evaluated. At 24 h after introducing siRNA against HNF4α (siHNF4a) or C/EBPα (siC/EBPa) or negative control (siCont), cells were transfected with pUC-HB-Ce or pGLHBp1627/1817 together with the LUC7L3-expressing plasmid (LUC7) or an empty vector (EV) and cultured for further 48 h. The reporter activities (upper left) and the pgRNA levels (upper right) were determined by the luciferase assay and RT-qPCR, respectively. Knockdown efficiency of HNF4α (lower left) and C/EBPα (lower right) was assessed by RT-qPCR. Results represent the means with SEM from three independent transfectants.

To identify the element within the ENII/BCP sequence responsible for the negative regulation of its transcriptional activity mediated by LUC7L3, a series of reporter constructs with partial ENII/BCP deletions were generated (Fig. 4A, left) and the reporter activity in the transfected cells with or without LUC7L3 expression was measured 2 days post-transfection. Although all deletions tested led to a decrease in the reporter activity, the deletion of nt 1666–1700 region (HBenIIcp-del-4) resulted in the complete loss of inhibitory effect by LUC7L3 (Fig. 4A, right). Interaction between LUC7L3 and the ENII/BCP region detected by DNA pull-down assay, in which the biotinylated ENII/BCP DNA probe can be complexed with LUC7L3 in the nuclear extract, was cancelled by deleting nt 1666–1700 sequence in the probe (ENIIBCP-del4 DNA) (Fig. 4B). However, the electrophoretic mobility shift assay based on *in vitro* synthesized LUC7L3 and a series of oligonucleotide probes resulted in no specific signal for the direct LUC7L3-DNA binding (Supplementary Fig. S2). It is thus likely that LUC7L3 recognizes indirectly the ENII/BCP presumably through nt 1666–1700 region. There are potential binding sites for HNF4α and C/EBPα, which are conserved among HBV genotypes, within nt 1666–1700 region. These transcription factors are known to function as positive regulators for HBV pregenome expression^2^,^4^. One may hypothesize that LUC7L3 suppresses ENII/BCP activity via possibly interacting either with HNF4α or C/EBPα. We addressed this possibility by knocking-down either HNF4α or C/EBPα, followed by introducing pUC-HB-Bj or the ENII/BCP reporter plasmid together with or without LUC7L3-expression plasmid (Fig. 4C). Knockdown either of HNF4α or C/EBPα led to a significant decrease in the pgRNA level and the ENII/BCP activity. However, the inhibitory effect of LUC7L3 on the pgRNA level and the ENII/BCP activity was not influenced by the knockdown; the pregenome levels in the knockdown- and control cells, respectively, were reduced to 30% (siHNF4a), 29% (siC/EBPa) and 26% (siCont) by LUC7L3 expression. LUC7L3 might regulate the ENII/BCP activity negatively by its interacting with certain transcription activator unidentified to date.

### Possible involvement of LUC7L3 in the regulation of pgRNA splicing

**Figure 5:**
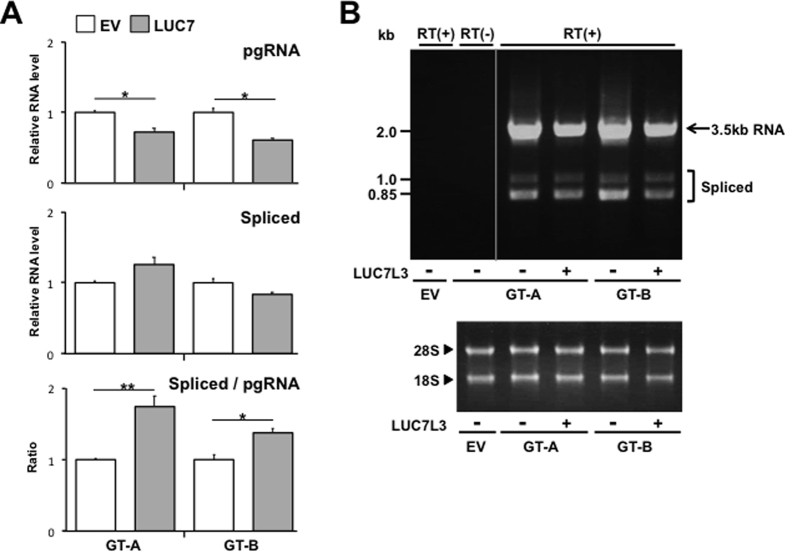
Effect of LUC7L3 expression on splicing of pgRNA. (**A**) HuH7 cells were transfected with the LUC7L3-expression plasmid (LUC7L3) or an empty vector (EV) together with either pUC-HB-Ae (GT-A), or -Bj (GT-B). At 48 h post-transfection, total RNAs were prepared from cells and subjected to RT-qPCRs for 3.5-kb pgRNA and pgRNA-derived spliced RNAs separately. Results represent the means with SEM from three independent transfectants. (**B**) Total RNAs obtained from the transfected cells as indicated above were used for semi-quantitative RT-PCR. cDNA bands corresponding to unspliced 3.5 kb pgRNA as well as several spliced forms including major spliced RNA derived from pgRNA were detected by agarose gel electrophoresis. The 28S and 18S ribosomal RNAs were also detected. Statistical significances compared with EV were shown. *p < 0.05, **p < 0.01, Student’s t test.

Since LUC7L3 is known to be a component of the U1 snRNP and is involved in the pre-mRNA splicing, whether LUC7L3 modulates splicing of HBV pregenome RNA was assessed by RT-qPCR (Fig. 5A) and semi-quantitative RT-PCR (Fig. 5B). After 48 h of co-transfection with pUC-HB-Ae or -HB-Bj and the LUC7L3-expression plasmid, total cellular RNAs were isolated and HBV spliced RNAs derived from pregenome, such as most abundant 2.2 kb singly-spliced RNA that lacks intron nt 2447/489, and unspliced, 3.5-kb pgRNA were separately determined. In RT-qPCR assay, the levels of unspliced pgRNA were lower in cells transfected with the LUC7L3-expression plasmid compared to control cells (Fig. 5A, top), analogous to Fig. 2E. By contrast, no or a little change in the spliced RNA levels was found in the LUC7L3-expressing cells (Fig. 5A, middle). As a result, over-expression of LUC7L3 led to increase in ratios of the spliced RNAs/unspliced pgRNA in HBV genotypes A and B (Fig. 5A, bottom). Such changes in the spliced/unspliced RNA ratios were confirmed by agarose gel electrophoresis of the corresponding cDNAs (Fig. 5B).

Thus, in addition to the inhibitory effect on the viral ENII/BCP activity, positive effect on the RNA splicing may be involved in negative regulation of the pgRNA level mediated by LUC7L3 during HBV lifecycle.

## Discussion

LUC7L3 is the human homologue of yeast splicing factor Luc7p, a component of the yeast U1 snRNP. This is categorized in the SR protein family and contains the arginine/serine-rich RS domain, which is generally located at the C terminus of pre-mRNA splicing factors and plays a role in splicing activation or alternative splicing regulation by engaging in protein-protein interactions^16^,^17^. It has been reported that LUC7L3 is possibly involved in alternative splicing of some cellular genes such as proapoptotic Bcl-x^18^ and a sodium channel member SCN5A^19^ through its association with another splicing factor RBM25. Besides the splicing regulation, only limited evidence for biological roles of LUC7L3 is available to date. LUC7L3 has also been demonstrated as cisplatin resistance-associated protein, cloned from cisplatin resistant cell lines by differential display^20^,^21^. Independently, LUC7L3 or its closely related human protein has been identified as a protein binding to the cAMP response element (CRE) by the yeast one-hybrid system^22^. However, the functional roles in the drug resistance or as the CRE-binding protein have not been elucidated.

In this study, among nuclear proteins capable for interacting with the ENII/BCP region of HBV genome as identified by the proteomics screening (Fig. 1A), we found that LUC7L3 functions as a negative regulator of HBV replication (Fig. 2). In addition to involvement in the pgRNA splicing (Fig. 5), LUC7L3 inhibited expression of pgRNA through down-regulation of the ENII/BCP activity (Figs 2B and 4A). Further, LUC7L3 expression decreased the levels of the viral cccDNA in cells (Fig. 3C) and of the particle-associated HBV DNA in the culture supernatants (Fig. 2E), indicating decrease in the viral replication induced by LUC7L3. The lower level of HBV replication potentially led to decrease in expression of whole viral antigens including HBc and HBs antigens (Fig. 2E). Deletion mutation analysis revealed that the C3H-type zinc finger motif at the N terminus of LUC7L3 is important for its negative regulation of the transcriptional activity. However, despite the fact that the motif is commonly found as a DNA binding motif in eukaryotic transcription factors or in RNA-binding proteins^23^,^24^,^25^, no direct binding between LUC7L3 and the ENII/BCP sequence was observed in the *in vitro* gel shift assay (Supplementary Fig. S2). Considering the results obtained in this study, a likely scenario is that the negative regulation of the ENII/BCP activity by LUC7L3 includes its interaction with yet-unidentified, positive regulator(s) bound to the ENII/BCP region, thus modulating the transcriptional activity. Recently, structural maintenance of chromosome complex SMC5/6 has been identified as a restriction factor for HBV gene expression^26^. We assessed effect of knockdown or over-expression of LUC7L3 on gene expression of SMC5 and SMC6 (Supplementary Fig. S3). Although LUC7L3 knowckdown redulted in a moderate increase in SMC5 and SMC6 mRNAs, over-expression of LUC7L3 exhibited no impact on expression of SMC5 and SMC6. Besides the cases with DNA/RNA binding, SR proteins are known to interact with RNA polymerase II or to act as cofactor to transcription factors such as CREB or AP-1^27^,^28^. Alternatively, LUC7L3 may contribute to down-regulation of expression of such positive regulator(s) through transcriptional or post-transcriptional process. Although further analysis to understand the mechanism underlying regulation of the LUC7L3-mediated ENII/BCP activity is needed, this study is the first to demonstrate the involvement of LUC7L3 in the transcriptional regulation.

It has been demonstrated that certain RNA splicing factors are involved in transcriptional regulation via interacting with transcription factors and/or basal transcriptional machinery. For example, PSF and p54nrb, in addition to their roles in facilitating RNA splicing, have been shown to function as transcriptional repressors for several nuclear receptors including progesterone receptor and androgen receptor^29^. Further study has indicated that PSF plays a role in repression of STAT6-mediated transcription through recruitment of the HDAC complex^30^. It has been reported that splicing factors such as PGC-1, CoAA, and CAPERs potentially coactivate the transcriptional activity of nuclear receptors^31^,^32^,^33^. Although for many years it has been thought that transcription and splicing are independent events, evidence to support the existence of functional links between these two processes is accumulating. Both transcription and pre-mRNA splicing are extremely complex steps where certain molecular interactions and kinetic constraints might be relevant. To understand functional roles of relatively poorly characterized LUC7L3 in the transcription/RNA processing machineries and their regulatory mechanisms, comprehensive analyses to identify a variety of interactions of LUC7L3 with the DNA/RNA sequences derived from viral and cellular target genes in the presence or absence of nuclear protein fractions should be required.

Among a number of host-cell factors that have been identified to regulate the HBV transcriptional activities via interactions with the ENII/BCP region, the majority are activators that stimulate the activities of the *cis*-acting elements. For example, liver-enriched or ubiquitous transcription factors such as C/EBPα, RXR, PPAR, HNF4, HNF3, FTF/LRH-1, TBP, FXRα, PGC-1α, SIRT1 and Sp1^2^,^4^ can bind to the ENII and contribute to the up-regulation of the core promoter activity. Regarding mechanisms for transcriptional repression of HBV gene expression, a negative regulatory element (NRE) that is located immediately upstream of ENII is known to be involved in down-regulation of the core promoter activity in an orientation-independent manner^2^,^4^. In addition, ENII, although less widely reported, is involved in negative regulation of the activity. Prox1, which is known as a corepressor of FTF/LRH-1, inhibits FTF/LRH-1-mediated activation of ENII^34^. IL-4 suppresses the core promoter activity presumably through down-regulation of C/EBPα expression^35^. It has also shown that TRIM proteins and COUP-TF1 potentially contribute to inhibition of ENII activity^36^,^37^. LUC7L3, a member of SR protein family, is a novel type of ENII-mediated negative regulator of HBV replication.

The study provided evidence valuable for understanding the HBV-host cell interaction during the viral replication cycle. Further studies to elucidate the molecular mechanism underlying LUC7L3-mediated suppression of HBV replication may provide new information on antiviral strategies.

## Methods

### Plasmids

Plasmids containing HBV genomes; pUC-HB-Ae, pUC-HB-Bj, and pUC-HB-CAT^38^, were gifts from Dr. Mizokami (National Center for Global Health and Medicine, Japan). DNA fragments of HBV core promoter derived from genotypes A and B were designed in accordance with the most common nucleotide among genotypes Ae and Bj, respectively, and synthesized (Eurofins Genomics, Ebersberg, Germany). To construct pGL4.10-HBpg-Ae/Bj, the synthesized fragments corresponding to nt 900–1817 region of HBV genome digested by KpnI and HindIII were inserted into pGL4.10 luciferase reporter (Promega, CA, USA). To construct pGL4.10-HBenIIcp-Ae/Bj, the synthesized fragments of nt 1627–1817 were amplified by PCR and digested by KpnI and HindIII, followed by being inserted into pGL4.10. A series of deletion mutants, pGL4.10-HBenIIcp-Bj-del1/del2/del3/del4/del5/del6/del7/del8/del9/del10 were generated based on pGL4.10- HBenIIcp-Bj. To create LUC7L3-expression plasmid pCAG-Flag-LUC7L3, human LUC7L3 sequence (Gene ID: 51747) was amplified by PCR using cDNA derived from HuH7 cells as a template, followed by digesting with BamHI and NotI and inserted into pCAG-Neo (Wako, Osaka, Japan). Parts of LUC7L3 gene were deleted in pCAG-Flag-LUC7L3, resulting in pCAG-Flag-LUC7L3-del1/del2/del3/del4/del5/del6. To create HNF4α-expression plasmid, the HNF4α sequence (Gene ID: 3172) was amplified by PCR as above, digested by EcoRI, and inserted into pCAGGS. Expression plasmids for C/EBPα and C/EBPβ were generated previously^39^.

### Cell culture, transfection, RNA interference and cell viability assay

HuH7 and 293T cells were maintained in Dulbecco modified Eagle medium with 10% fetal bovine serum. Cells (1 × 10^5^ cells/well in a 12-well plate) were transiently transfected with 0.5 μg of plasmid DNA mixed with Lipofectamine LTX (Invitrogen, CA, USA). ACIN1-specific siRNA, BCLAF1-specific siRNA, CNBP1-specific siRNA, CTCF-specific siRNA, HDGFRP2-specific siRNA, HMGN1-specific siRNA, LUC7L3-specific siRNA (siLUC7) and the negative control RNA (siNC) was provided by Bonac (Fukuoka, Japan). siRNAs for HNF4α (siHNF4a), C/EBPα (siC/EBPa) and the negative control RNA (siNC-2) were purchased from Ambion (CA, USA). The synthetic siRNAs (50 pmol) were transfected into cells using ScreenFect A (Wako). Total RNAs and proteins of cells were prepared at 72 h post-transfection. Cell viability was measured by using a CellTiter-Glo luminescent cell viability assay (Promega).

### Immunoblotting and immunocytochemistry

Immunoblotting and immunocytochemistry were preformed essentially as described^40^. Cell lyses with 1% NP-40, 0.1% SDS, 1% sodium deoxycholate, 25 mM Tris-HCl, pH 7.6, 150 mM NaCl, 1 mM EDTA, protease inhibitor cocktail (Roche Diagnostics, Switzerland), were separated by SDS–PAGE and transferred onto PVDF membranes. After blocking for 1 h, the membranes were incubated with an antibody against HBc, which was generated by immunizing rabbits with bacterially-expressed HBc protein, HBs (Institute of Immunology, Tokyo, Japan), LUC7L3 (SIGMA Aldrich), HNF4α (Santa Cruz Biotechnology, Texas, USA), C/EBPα (Santa Cruz Biotechnology), FLAG M2 (Sigma-Aldrich, Tokyo, Japan) or GAPDH (Santa Cruz Biotechnology) for 1 h. After washing, membranes were incubated with HRP-conjugated secondary antibody (Cell Signaling, MA, USA) for 0.5–1 h. Antigen-antibody complexes were detected using the ECL prime Western blotting Detection Reagent (GE Healthcare, Bucks, UK). For immunocytochemistry, cells grown on a glass bottom plate were fixed with 4% paraformaldehyde (Wako) for 15 min and permeabilized in 0.5% Triton X-100 in PBS, followed by blocking with 1% bovine serum albumin. Immunocytochemistry was performed with the anti-LUC7L3 antibody (Sigma Aldrich) for 2 h, followed by Alexa Fluor 488 anti-rabbit IgG (H + L) antibody (Vector Laboratories, CA, USA) for 2 h. Double-stranded DNA was stained with Hochest 33342 (Dojin, Tokyo, Japan). Subcellular localization of LUC7L3 was observed under the confocal microscope FV1000-D (Olympus, Tokyo, Japan).

### Luciferase reporter assay

Cells were transiently co-transfected with the firefly luciferase reporter and pGL4.75 carrying *Renilla* luciferase gene with CMV promoter (Promega), which was used for normalizing transfection efficiency. At 48 h post-transfection, luciferase activities in cell lysates were measured with the dual luciferase reporter assay kit (Promega).

### Quantification of DNA and RNA of HBV

To determine HBV pgRNA and spliced RNAs derived from pgRNA, total RNAs were extracted from transfected cells with TRI-reagent (MRC, OH, USA) according to the manufacture’s instruction. The RNAs were treated with DNase I (TaKaRa, Shiga, Japan) and RNase inhibitor (TaKaRa). cDNA templates were synthesized using SuperScript VILO cDNA synthesis kit (Invitrogen), and qPCR was carried out using the SYBR qPCR Mix kit (Toyobo, Osaka, Japan) with the following primer sets; 5′-TCCCTCGCCTCGCAGACG-3′ and 5′-GTTTCCCACCTTATGAGTC-3′ for unspliced, 3.5-kb pgRNA, and 5′-CCGCGTCGCAGAAGATCT-3′ and 5′-CTGAGGCCCACTCCCATAGG-3′ for spliced RNAs derived from pgRNA. The GAPDH gene was adopted as an internal standard for the assay and the primers; 5′-AACAGCCTCAAGATCATCAGC-3′ and 5′-GGATGATGTTCTGGAGAGCC-3′ were used. For semi-quantitative RT-PCR, the cDNA templates were amplified with the following primers, 5′-AGCCTCCAAGCTGTGCCTTGGGTG-3′ and 5′-AACCACTGAACAAATGGCACTAGTAAACTGAGC-3′. Agarose gel electrophoresis was used for the separation of PCR products corresponding to unspliced and spliced forms of pgRNA.

To quantify the particle-associated HBV DNA, culture supernatants collected were treated with PNE buffer (8.45% PEG, 0.445 M NaCl, 13 mM EDTA) on ice for 1 h. After centrifugation at 12000 rpm for 15 min, the pellets were treated with DNase I and RNase for 1 h at 37 °C to remove free nucleic acids. The samples were then lysed with the lysis buffer (13.33 mM Tris-HCl, 6.6 mM EDTA, and 0.67% SDS) containing proteinase K (Wako) at 56 °C overnight. DNAs in the lysates were isolated by phenol/chloroform extraction and ethanol precipitation. The viral DNA was then analyzed by qPCR with primers used for the analysis of unspliced pgRNA as indicated above.

### mRNA quantification of cellular genes

Expression of LUC7L3 mRNA in various tissue was examined by using the multiple tissue cDNA (MTC) human panels I and II (Clontech laboratories, CA, USA). The panels contained a set of normalized single-strand cDNAs, produced from poly(A)+ RNA from various normal human tissues. qPCRs were performed using the SYBR qPCR Mix kit (Toyobo, Osaka, Japan). Expression of LUC7L3 mRNA was standardized by that of RNA polymerase II (RPII) gene. The primers used were 5′-TCAAGCCGAACATCAGACAG-3′ and 5′-GCTTCTGCTTCTTCGTCGAT-3′ for LUC7L3 and 5′-GCACCACGTCCAATGACAT-3′ and 5′-GTGCGGCTGCTTCCATAA-3′ for RPII. For RT-qPCR to determine mRNAs of SMC5 and SMC6, total cellular RNAs isolated by TRI reagent were transcribed using SuperScript VILO cDNA Synthesis Kit. Aliquots of cDNAs were subjected to 45 cycles of PCR amplification using THUNDERBIRD SYBR qPCR mix. The primers used were 5′-AGAAGCAAGATGTTATAGAAAGGAAAG-3′ and 5′-TCCTCTGTCGGTCAAGCTCT-3′ for SMC5 and 5′-TGCATCAATTCTGGACAAAGA-3′ and 5′- TGCTTCTTGGTACTGCCTCA-3′ for SMC6. The RNA expression data were normalized to levels of reference gene GAPDH using the comparative threshold cycle method.

### Southern blot analysis

Extraction of protein-free DNAs that contain cccDNA and protein-free relaxed circular (rc)DNA was carried out by using a modified Hirt extraction procedure^41^,^42^. Briefly, cells collected from a 10 cm diameter dish were lysed in 3 ml of 10 mM Tris-HCl (pH 7.5), 10 mM EDTA, and 0.7% SDS. After 30 min incubation at room temperature, the lysate was transferred into a 15-ml tube, followed by addition of 0.8 ml of 5 M NaCl and incubation at 4 °C overnight. The lysate was then clarified by centrifugation at 12,000 × g for 30 min at 4 °C and extracted twice with phenol and once with phenol:chloroform. The resulting DNA was precipitated with two volumes of ethanol overnight at room temperature. 10 μg of the protein-free DNA sample was resolved in a 1.2% agarose gel and transferred onto Hybond-XL membrane. Detection of HBV DNA was performed using the DIG RNA Labeling Mix (Roche Diagnostics). Membranes were probed with a DIG-labeled 0.4 kb HBV RNA probe corresponding to nt 1998–2453 (GenBank. No. AB246338). Hybridization was carried out in 5 ml of DIG EASY Hyb reagent (Roche Diagnostics) at 68 °C overnight, followed by washing with 0.1× SSC and 0.1% SDS at 68 °C. The membrane was blocked with blocking solution for 30 min, and incubated with anti-DIG antibody (Roche Diagnostics) for 1 h. CDP-star (Roche Diagnostics) was used as a substrate and hybridization signals on the membrane were detected by ChemiDoc Touch Gel Imaging System (Bio-Rad Laboratories, Tokyo, Japan).

### Detection of DNA-protein interactions

Nuclear proteins that interact with the DNA sequence corresponding to the ENII/BCP (nt 1627–1817) were isolated using FactorFinder Kit (Miltenyi Biotec, Bergisch Gladbach, Germany) according to the manufacturer’s instructions. The biotin-labeled capture ENII/BCP DNA probe was generated by PCR with biotinylated primers; 5′-CGTGAACGCCCACCGGAACC-3′ and 5′-GTTGCATGGTGCTGGTGAAC-3′. The streptavidin-conjugated microbeads were used to isolate DNA-protein complexes, followed by mass-spectrometry (MS) analysis to identify proteins using mass spectrometry Q Exactive Hybrid Quadrupole-Orbitrap Mass Spectrometer (Thermo fisher, CA, USA) with Xcalibur (version 2.2). The Proteome Discoverer software (version 1.4; Thermo Fisher Scientific) was used to generate peak lists from the raw MS data files. The resulting peak lists were subsequently submitted to a SEQUEST search engine (Thermo Fisher Scientific) and compared against the human protein sequences in the UniProt protein database (Reviewed-Human + [9606]-20130806; 70236 sequences) to identify peptides. The SEQUEST search parameters were as follows: variable modifications including oxidation of methionine and carbamidomethyl of cysteine; peptide mass tolerance of ±10 ppm; fragment mass tolerance of ±0.02 Da.

For detection of the DNA-LUC7L3 interaction, nuclear extracts obtained from cells transfected with a plasmid expressing FLAG-tagged LUC7L3 or its deletion mutant were used. The DNA-protein complexes were analyzed by immunoblotting with anti-FLAG antibody. To prepare ampicillin DNA probe as an unrelated capture DNA probe, PCR was performed using biotinylated primers; 5′-TTCGTTCGTCCATAGTGGCCTG-3′ and 5′-CATAGACTGGATGGAGGCGGAC-3′, and pGL4.10 (Promega) as a template.

## Additional Information

**How to cite this article:** Li, Y. *et al*. LUC7L3/CROP inhibits replication of hepatitis B virus via suppressing enhancer II/basal core promoter activity. *Sci. Rep.* **6**, 36741; doi: 10.1038/srep36741 (2016).

**Publisher’s note:** Springer Nature remains neutral with regard to jurisdictional claims in published maps and institutional affiliations.

## Supplementary Material

Supplementary Information

## References

[b1] Locarnini S. Molecular virology of hepatitis B virus. Semin. Liver Dis 24 Suppl 1, 3–10 (2004). 10.1055/s-2004-828672 15192795

[b2] Quarleri J. Core promoter: a critical region where the hepatitis B virus makes decisions. World J Gastroenterol 20, 425–435 (2014). 24574711 10.3748/wjg.v20.i2.425 PMC3923017

[b3] Seeger C. & Mason W. S. Molecular biology of hepatitis B virus infection. Virology 479–480, 672–686 (2015). 10.1016/j.virol.2015.02.031 PMC4424072 25759099

[b4] Moolla N., Kew M. & Arbuthnot P. Regulatory elements of hepatitis B virus transcription. J Viral Hepat 9, 323–331 (2002). 12225325 10.1046/j.1365-2893.2002.00381.x

[b5] Gunther S. . Type, prevalence, and significance of core promoter/enhancer II mutations in hepatitis B viruses from immunosuppressed patients with severe liver disease. J Virol 70, 8318–8331 (1996). 8970951 10.1128/jvi.70.12.8318-8331.1996 PMC190919

[b6] Baumert T. F., Marrone A., Vergalla J. & Liang T. J. Naturally occurring mutations define a novel function of the hepatitis B virus core promoter in core protein expression. J Virol 72, 6785–6795 (1998). 9658127 10.1128/jvi.72.8.6785-6795.1998 PMC109887

[b7] Buckwold V. E., Xu Z., Chen M., Yen T. S. & Ou J. H. Effects of a naturally occurring mutation in the hepatitis B virus basal core promoter on precore gene expression and viral replication. J Virol 70, 5845–5851 (1996). 8709203 10.1128/jvi.70.9.5845-5851.1996 PMC190601

[b8] Tacke F. . Basal core promoter and precore mutations in the hepatitis B virus genome enhance replication efficacy of Lamivudine-resistant mutants. J Virol 78, 8524–8535 (2004). 15280461 10.1128/JVI.78.16.8524-8535.2004 PMC479060

[b9] Guarnieri M. . Point mutations upstream of hepatitis B virus core gene affect DNA replication at the step of core protein expression. J Virol 80, 587–595 (2006). 16378961 10.1128/JVI.80.2.587-595.2006 PMC1346833

[b10] Laskus T. . Association between hepatitis B virus core promoter rearrangements and hepatocellular carcinoma. Biochem Biophys Res Commun 244, 812–814 (1998). 9535748 10.1006/bbrc.1998.8249

[b11] Baptista M., Kramvis A. & Kew M. C. High prevalence of 1762(T) 1764(A) mutations in the basic core promoter of hepatitis B virus isolated from black Africans with hepatocellular carcinoma compared with asymptomatic carriers. Hepatology 29, 946–953 (1999). 10051502 10.1002/hep.510290336

[b12] Blackberg J. & Kidd-Ljunggren K. Mutations within the hepatitis B virus genome among chronic hepatitis B patients with hepatocellular carcinoma. J Med Virol 71, 18–23 (2003). 12858404 10.1002/jmv.10458

[b13] Kao J. H., Chen P. J., Lai M. Y. & Chen D. S. Basal core promoter mutations of hepatitis B virus increase the risk of hepatocellular carcinoma in hepatitis B carriers. Gastroenterology 124, 327–334 (2003). 12557138 10.1053/gast.2003.50053

[b14] Fortes P. . Luc7p, a novel yeast U1 snRNP protein with a role in 5′ splice site recognition. Genes Dev 13, 2425–2438 (1999). 10500099 10.1101/gad.13.18.2425 PMC317023

[b15] Puig O., Bragado-Nilsson E., Koski T. & Seraphin B. The U1 snRNP-associated factor Luc7p affects 5′ splice site selection in yeast and human. Nucleic Acids Res 35, 5874–5885 (2007). 17726058 10.1093/nar/gkm505 PMC2034479

[b16] Philipps D., Celotto A. M., Wang Q. Q., Tarng R. S. & Graveley B. R. Arginine/serine repeats are sufficient to constitute a splicing activation domain. Nucleic Acids Res 31, 6502–6508 (2003). 14602908 10.1093/nar/gkg845 PMC275541

[b17] Godin K. S. & Varani G. How arginine-rich domains coordinate mRNA maturation events. RNA Biol 4, 69–75 (2007). 17873524 10.4161/rna.4.2.4869

[b18] Zhou A., Ou A. C., Cho A., Benz E. J. Jr. & Huang S. C. Novel splicing factor RBM25 modulates Bcl-x pre-mRNA 5′ splice site selection. Mol Cell Biol 28, 5924–5936 (2008). 18663000 10.1128/MCB.00560-08 PMC2546994

[b19] Gao G. & Dudley S. C. Jr. RBM25/LUC7L3 function in cardiac sodium channel splicing regulation of human heart failure. Trends Cardiovasc Med 23, 5–8 (2013). 22939879 10.1016/j.tcm.2012.08.003 PMC3532530

[b20] Nishii Y. . CROP/Luc7A, a novel serine/arginine-rich nuclear protein, isolated from cisplatin-resistant cell line. FEBS Lett. 465, 153–156 (2000). 10631324 10.1016/s0014-5793(99)01744-5

[b21] Umehara H. . Effect of cisplatin treatment on speckled distribution of a serine/arginine-rich nuclear protein CROP/Luc7A. Biochem Biophys Res Commun 301, 324–329 (2003). 12565863 10.1016/s0006-291x(02)03017-6

[b22] Shipman K. L., Robinson P. J., King B. R., Smith R. & Nicholson R. C. Identification of a family of DNA-binding proteins with homology to RNA splicing factors. Biochem. Cell Biol 84, 9–19 (2006). 16462885 10.1139/o05-139

[b23] Baxevanis A. D. & Vinson C. R. Interactions of coiled coils in transcription factors: where is the specificity? Curr Opin Genet Dev 3, 278–285 (1993). 8504253 10.1016/0959-437x(93)90035-n

[b24] Laity J. H., Lee B. M. & Wright P. E. Zinc finger proteins: new insights into structural and functional diversity. Curr. Opin. Struct. Biol. 11, 39–46 (2001). 11179890 10.1016/s0959-440x(00)00167-6

[b25] Gamsjaeger R., Liew C. K., Loughlin F. E., Crossley M. & Mackay J. P. Sticky fingers: zinc-fingers as protein-recognition motifs. Trends Biochem Sci 32, 63–70 (2007). 17210253 10.1016/j.tibs.2006.12.007

[b26] Decorsière A. . Hepatitis B virus X protein identifies the Smc5/6 complex as a host restriction factor. Nature 531, 386–389 (2016). 26983541 10.1038/nature17170

[b27] Meruvu S., Hugendubler L. & Mueller E. Regulation of adipocyte differentiation by the zinc finger protein ZNF638. J Biol Chem 286, 26516–26523 (2011). 21602272 10.1074/jbc.M110.212506 PMC3143616

[b28] Long J. C. & Caceres J. F. The SR protein family of splicing factors: master regulators of gene expression. Biochem J 417, 15–27 (2009). 19061484 10.1042/BJ20081501

[b29] Dong X., Sweet J., Challis J. R., Brown T. & Lye S. J. Transcriptional activity of androgen receptor is modulated by two RNA splicing factors, PSF and p54nrb. Mol Cell Biol 27, 4863–4875 (2007). 17452459 10.1128/MCB.02144-06 PMC1951499

[b30] Dong L. . PTB-associated splicing factor (PSF) functions as a repressor of STAT6-mediated Ig epsilon gene transcription by recruitment of HDAC1. J Biol Chem 286, 3451–3459 (2011). 21106524 10.1074/jbc.M110.168377 PMC3030351

[b31] Knutti D. & Kralli A. PGC-1, a versatile coactivator. Trends Endocrinol Metab 12, 360–365 (2001). 11551810 10.1016/s1043-2760(01)00457-x

[b32] Dowhan D. H. . Steroid hormone receptor coactivation and alternative RNA splicing by U2AF65-related proteins CAPERalpha and CAPERbeta. Mol Cell 17, 429–439 (2005). 15694343 10.1016/j.molcel.2004.12.025

[b33] Brooks Y. S. . Functional pre- mRNA trans-splicing of coactivator CoAA and corepressor RBM4 during stem/progenitor cell differentiation. J Biol Chem 284, 18033–18046 (2009). 19416963 10.1074/jbc.M109.006999 PMC2709364

[b34] Qin J. . Prospero-related homeobox protein (Prox1) inhibits hepatitis B virus replication through repressing multiple cis regulatory elements. J Gen Virol 90, 1246–1255 (2009). 19264593 10.1099/vir.0.006007-0

[b35] Lin S. J., Shu P. Y., Chang C., Ng A. K. & Hu C. P. IL-4 suppresses the expression and the replication of hepatitis B virus in the hepatocellular carcinoma cell line Hep3B. J Immunol 171, 4708–4716 (2003). 14568946 10.4049/jimmunol.171.9.4708

[b36] Fischer S. F. . Genotype-dependent activation or repression of HBV enhancer II by transcription factor COUP-TF1. World J Gastroenterol 12, 6054–6058 (2006). 17009409 10.3748/wjg.v12.i37.6054 PMC4124418

[b37] Zhang S., Guo J. T., Wu J. Z. & Yang G. Identification and characterization of multiple TRIM proteins that inhibit hepatitis B virus transcription. PLoS ONE 8, e70001 (2013). 23936368 10.1371/journal.pone.0070001 PMC3731306

[b38] Sugiyama M. . Influence of hepatitis B virus genotypes on the intra- and extracellular expression of viral DNA and antigens. Hepatology 44, 915–924 (2006). 17006908 10.1002/hep.21345

[b39] Masaki T. . All-trans retinoic acid down-regulates human albumin gene expression through the induction of C/EBPbeta-LIP. Biochem J 397, 345–353 (2006). 16608438 10.1042/BJ20051863 PMC1513275

[b40] Suzuki R. . Signal peptidase complex subunit 1 participates in the assembly of hepatitis C virus through an interaction with E2 and NS2. PLoS Pathog 9, e1003589 (2013). 24009510 10.1371/journal.ppat.1003589 PMC3757040

[b41] Hirt B. Selective extraction of polyoma DNA from infected mouse cell cultures. J Mol Biol 26, 365–369 (1967). 4291934 10.1016/0022-2836(67)90307-5

[b42] Cai D. . A southern blot assay for detection of hepatitis B virus covalently closed circular DNA from cell cultures. Methods Mol Biol 1030, 151–161 (2013). 23821267 10.1007/978-1-62703-484-5_13 PMC5060941

