# Supplementary material for: LUC7L3/CROP inhibits replication of hepatitis B virus via suppressing enhancer II/basal core promoter activity
Source: Sci Rep. 2016 Nov 18;6:36741. doi: 10.1038/srep36741 (PMC5114668; doi:10.1038/srep36741)
Supplement: Supplementary Information [file srep36741-s1.pdf]

LUC7L3/CROP inhibits replication of hepatitis B virus via suppressing enhancer II/basal core promoter activity

Yuan Li[1], Masahiko Ito[1], Suofeng Sun[1], Takeshi Chida[1], Kenji Nakashima[1], Tetsuro Suzuki[1]

[1]Department of Virology and Parasitology, Hamamatsu University School of Medicine, Shizuoka 431-3192, Japan

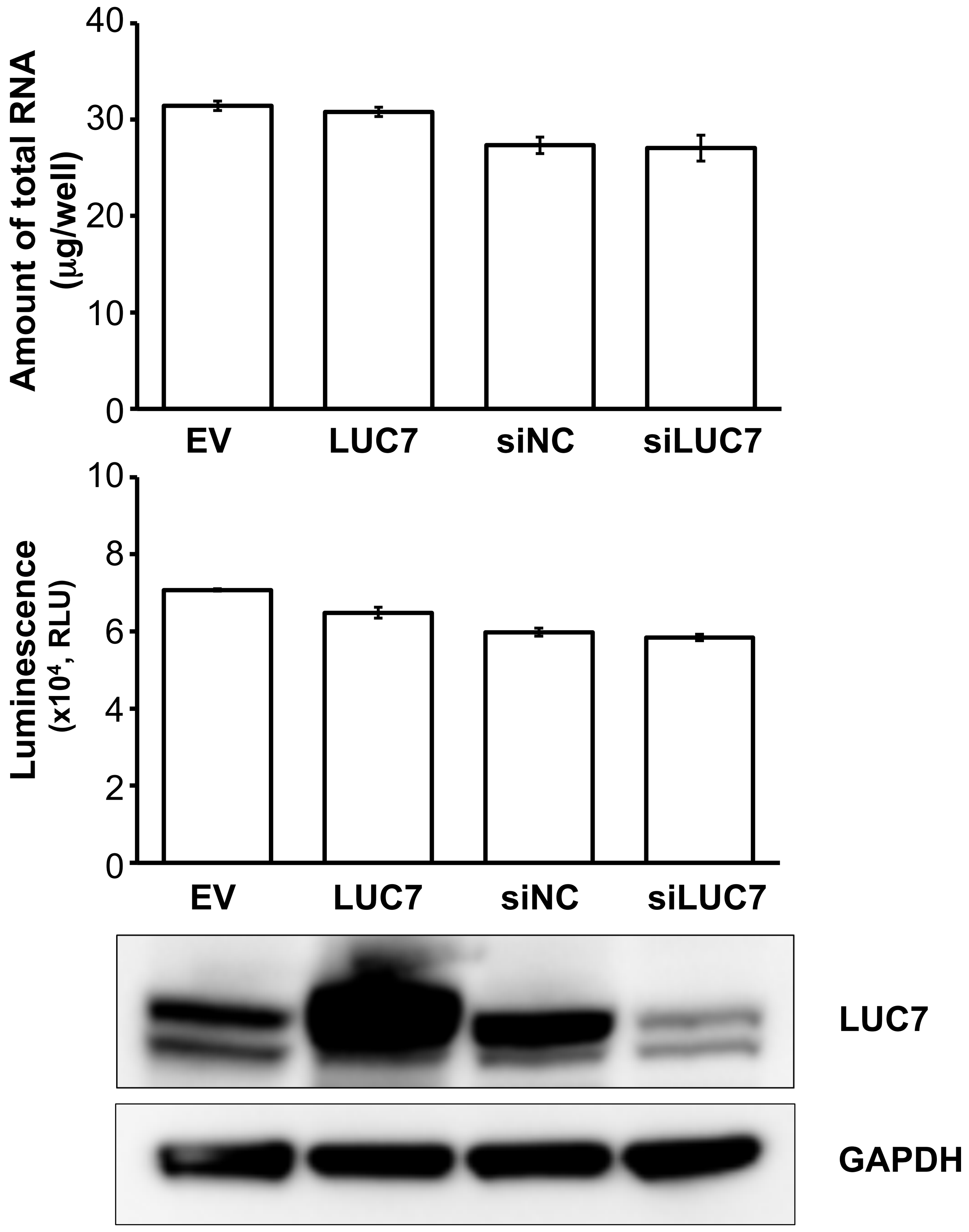

Supplementary Figure S1

Effect of over-expression or knockdown of LUC7L3 on the cell viability. For knockdown of LUC7L3, HuH7 cells were transfected with siRNAs (siLUC7) or its negative control (siNC), and harvested 4 days later. For over-expression of LUC7L3, HuH7 cells were transfected with the LUC7L3-expression plasmid (LUC7) or an empty vector (EV), and harvested 2 days later. For the analysis of cell viability, amounts of total cellular RNAs were determined (upper) and ATP contents in cells were measured by CellTiter-Glo luminescent cell viability assay kit (middle). Protein expression of LUC7L3 as well as of GAPDH was assessed by immunoblotting (lower). Values shown represent the means with SEM obtained from three independent transfection of siRNAs or plasmids.

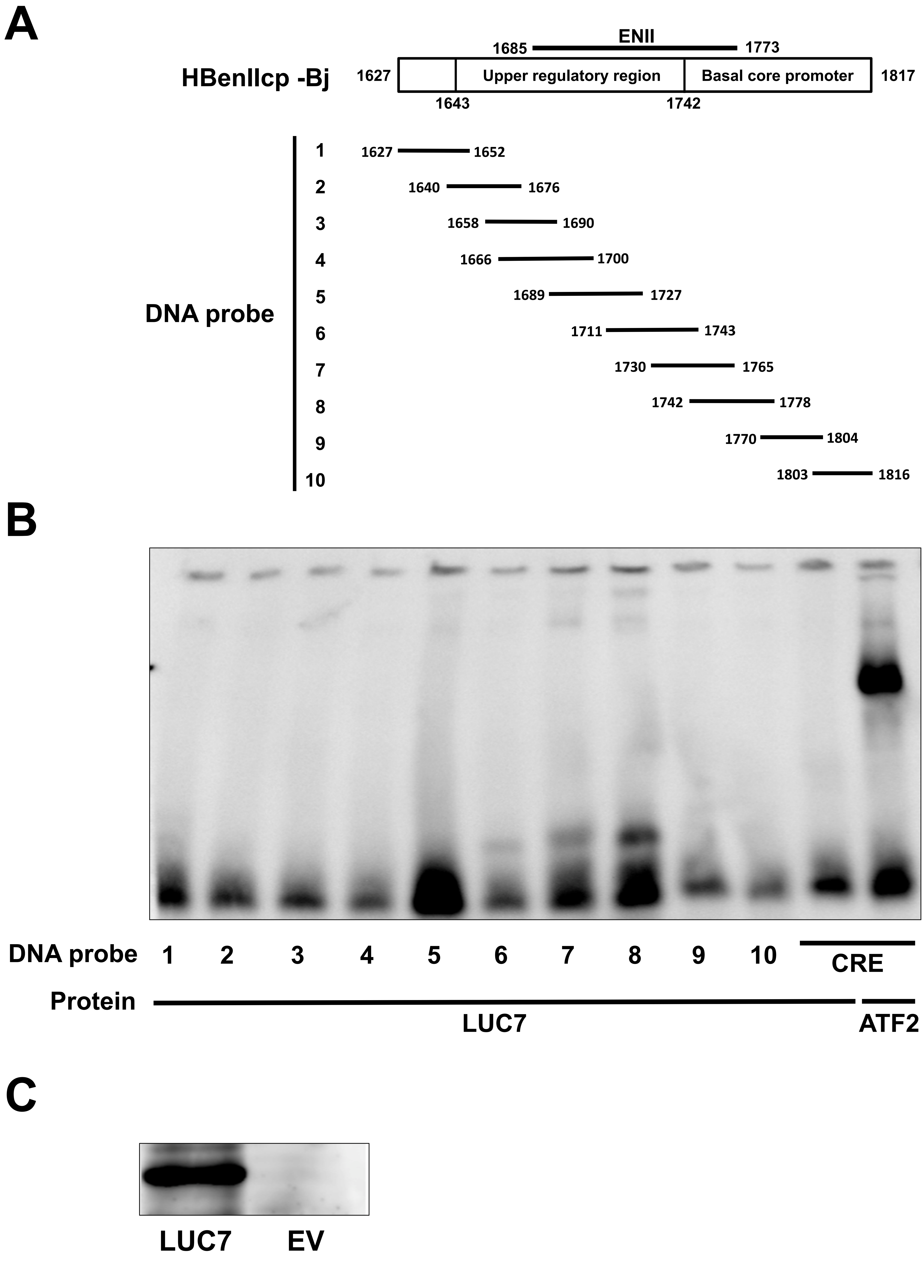

Supplementary Figure S2

The electrophoretic mobility shift assay based on in vitro synthesized LUC7L3. A series of DNA probes for ENII/BCP region of the HBV genome used in this study were indicated (A). Aliquots of in vitro synthesized LUC7L3 or ATF2 were mixed with a probe shown in (A), leading to the gel shift assay. Consensus binding sequences of CRE were used as probes for positive controls (B). In vitro translation of LUC7L3 was confirmed by immunoblotting (C).

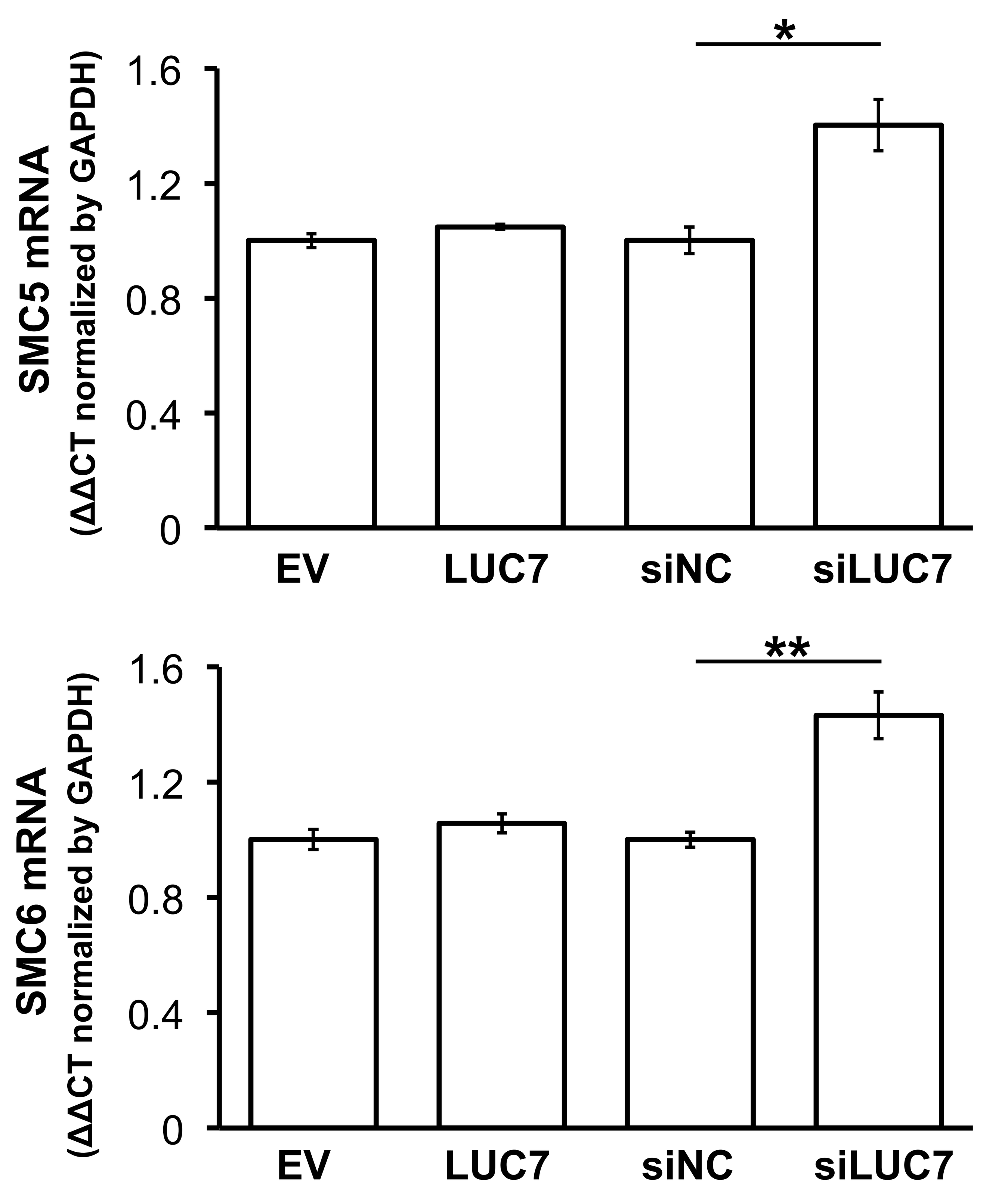

Supplementary Figure S3

Effect of LUC7L3 on mRNA expression of SMC5 and SMC6. After overexpression or knockdown of LUC7L3, mRNA level of SMC5 (upper) and SMC6 (lower) was determined by qRT-PCR. For over-expression of LUC7L3, HuH7 cells were transfected with the LUC7L3-expression plasmid (LUC7) or an empty vector (EV), and harvested 2 days later. For knockdown of LUC7L3, HuH7 cells were transfected with siRNAs (siLUC7) or its negative control (siNC), and harvested 4 days later. Values shown represent the means with SEM obtained from three independent transfection of siRNAs or plasmids. Statistical significances compared with EV or siNC were shown. *$p<0.05$, **$p<0.01$, Student’s t test.

Supplementary Table S1. Proteins pulled down by ENII/BCP (nt 1627-1817) DNA probe.

Accession*	Gene name	Score**	Accession*	Gene name	Score**
P16403	HIST1H1C	1992.92	O75531	BANF1	77.54
P10412	HIST1H1E	1837.35	P05114	HMGN1	75.35
P16401	HIST1H1B	1809.89	P68104	EEF1A1	74.32
P16402	HIST1H1D	1390.47	Q15287	RNPS1	73.89
Q02539	HIST1H2BC	875.52	P38646	HSPA9	71.78
P19338	NCL	632.71	Q13151	HNRNPA0	69.72
P35579	MYH9	592.47	P18124	RPL7	66.50
P62805	HIST1H4A	534.35	P25705	ATP5A1	60.05
**Q9UKV3**	**ACIN1**	**513.33**	P49756	RBM25	57.77
P62807	HIST1H2B1C	503.80	Q8IYB3	SRRM1	54.91
Q99880	HIST1H2BL	480.66	P05204	HMGN2	53.87
P60709	ACTB	478.00	P11387	TOP1	52.45
P62750	RPL23A	472.65	P52926	HMGA2	50.38
Q16778	HIST2H2BE	464.26	Q07666	KHDRBS1	48.55
P06899	HIST1H2BJ	446.49	P31943	HNRNPH1	47.81
P20671	HIST1H2AD	428.68	Q04837	SSBP1	45.26
Q8N257	HIST3H2BB	423.85	O00479	HMGN4	45.06
Q93077	HIST1H2AC	417.48	P62841	RPS15	43.56
P04908	HIST1H2AB	399.76	Q9NR30	DDX21	43.42
Q00839	HNRNPU	378.58	Q8NFC6	BOD1L1	42.65
P07305	H1F0	375.95	Q15365	PCBP1	40.90
Q02878	RPL6	356.03	P08621	SNRNP70	40.16
P67809	YBX1	282.77	P04406	GAPDH	40.01
Q14978	NOLC1	276.67	O00571	DDX3X	39.88
P12956	XRCC6	262.29	**Q9NYF8**	**BCLAF1**	**39.38**
Q92522	H1FX	259.89	Q9UKM9	RALY	38.29
Q5SSJ5	HP1BP3	231.42	**O95232**	**LUC7L3**	**37.62**
P68431	HIST1H3A	177.13	P62847	RPS24	36.04
P35580	MYH10	174.06	P42677	RPS27	35.57
P68363	TUBA1B	166.56	Q05519	SRSF11	34.70
Q9BQE3	TUBA1C	164.32	Q14980	NUMA1	34.28
O75683	SURF6	156.78	**P17096**	**HMGA1**	**33.58**
P07437	TUBB	140.16	Q9UHX1	PUF60	33.43
P07910	HNRNPC	125.75	**P62633**	**CNBP1**	**33.35**
P0C0S5	H2AFZ	121.36	**Q7Z4V5**	**HDGFRP2**	**30.11**
P11021	HSPA5	119.97	O95831	AIFM1	29.58
P11142	HSPA8	118.80	O60841	EIF5B	29.45
Q9H307	PNN	109.13	P52272	HNRNPM	26.03
P13010	XRCC5	97.94	P10809	HSPD1	25.45
Q9HCC0	MCCC2	88.72	P23396	RPS3	24.09
P68371	TUBB4B	83.90	Q92466	DDB2	23.63
P42696	RBM34	83.15	Q92841	DDX17	22.82
O94776	MTA2	82.36	Q8WUU4	ZNF296	22.45
P45880	VDAC2	78.79	**P49711**	**CTCF**	**20.18**
P38159	RBMX	78.02			

Note: Genes analyzed in this study are highlighted in bold. *Swiss-Prot/UniProtKB accession number. ** Sum of the scores of the individual peptides calculated by SEQUEST algorithm for the match.
